# The Development of Hyperspectral Distribution Maps to Predict the Content and Distribution of Nitrogen and Water in Wheat (*Triticum aestivum*)

**DOI:** 10.3389/fpls.2019.01380

**Published:** 2019-10-30

**Authors:** Brooke Bruning, Huajian Liu, Chris Brien, Bettina Berger, Megan Lewis, Trevor Garnett

**Affiliations:** ^1^Australian Plant Phenomics Facility, The Plant Accelerator, School of Agriculture, Food & Wine, University of Adelaide, Urrbrae, SA, Australia; ^2^Ecology and Evolutionary Biology, School of Biological Sciences, University of Adelaide, Adelaide, SA, Australia

**Keywords:** nitrogen, water, hyperspectral, wheat, PLSR, plant phenotyping

## Abstract

Quantifying plant water content and nitrogen levels and determining water and nitrogen phenotypes is important for crop management and achieving optimal yield and quality. Hyperspectral methods have the potential to advance high throughput phenotyping efforts by providing a rapid, accurate, and nondestructive alternative for estimating biochemical and physiological plant traits. Our study (i) acquired hyperspectral images of wheat plants using a high throughput phenotyping system, (ii) developed regression models capable of predicting water and nitrogen levels of wheat plants, and (iii) applied the regression coefficients from the best-performing models to hyperspectral images in order to develop prediction maps to visualize nitrogen and water distribution within plants. Hyperspectral images were collected of four wheat (*Triticum aestivum*) genotypes grown in nine soil nutrient conditions and under two water treatments. Five multivariate regression methods in combination with 10 spectral preprocessing techniques were employed to find a model with strong predictive performance. Visible and near infrared wavelengths (VNIR: 400–1,000nm) alone were not sufficient to accurately predict water and nitrogen content (validation R^2^ = 0.56 and R^2^ = 0.59, respectively) but model accuracy was improved when shortwave-infrared wavelengths (SWIR: 1,000–2,500nm) were incorporated (validation R^2^ = 0.63 and R^2^ = 0.66, respectively). Wavelength reduction produced equivalent model accuracies while reducing model size and complexity (validation R^2^ = 0.69 and R^2^ = 0.66 for water and nitrogen, respectively). Developed distribution maps provided a visual representation of the concentration and distribution of water within plants while nitrogen maps seemed to suffer from noise. The findings and methods from this study demonstrate the high potential of high-throughput hyperspectral imagery for estimating and visualizing the distribution of plant chemical properties.

## Introduction

Wheat (*Triticum aestivum*) is the major winter crop in Australia and sustainable improvement of yields is a major research focus. The availability of nitrogen and water are widely recognized as two of the main factors limiting crop growth and production (Garnett and Rebetzke, 2013). Nitrogen is essential for crops but nitrogen use efficiency is generally low (Raun and Johnson, 1999). Understanding the nitrogen dynamics within the plant is key to improving fertilization practices and breeding more efficient crops (Garnett et al., 2015) which in turn may help reach the increased yields required for a growing population. Water also profoundly influences plant health and potential yield. Water is a strong driver of photosynthesis, respiration, absorption, and the translocation of nutrients and metabolites throughout the plant (Lambers et al., 2008). The accurate assessment of water content also has importance for fertilization, irrigation practices, and drought assessment (Peñuelas et al., 1997; Torres et al., 2019).

High throughput plant phenotyping is an emerging approach for plant breeding and crop improvement studies (Awada et al., 2018; Hansen et al., 2018). Image-based phenotyping offers nondestructive techniques which can significantly reduce the cost, time, and labor involved in larger-scale screening trials. Collecting information from multiple sensors allows near-simultaneous data collection for the measurement of many plant traits (Li et al., 2014; Humplik et al., 2015). Since such methods are nondestructive, they are repeatable across a plant’s lifecycle, thereby allowing for changes to be detected over time which otherwise could not be determined with traditional destructive analyses (Berger et al., 2012; Fahlgren et al., 2015).

Hyperspectral cameras are becoming more common in the plant research environment. Hyperspectral imaging combines the benefits of both spectroscopy and traditional imaging; it is able to quantify light reflectance across hundreds of narrow spectral bands for distinct spatial pixels (ElMasry et al., 2012; Liu et al., 2014). Hyperspectral imaging has successfully been used in the prediction of many plant traits such as early drought stress in barley (Behmann et al., 2014), macronutrient content and distribution in oilseed rape (Zhang et al., 2013), and nitrogen distribution in cucumber leaves (Yu et al., 2014). Chemical properties, including water content, micronutrient, and macronutrient concentrations, have also been quantified in maize and soybean plants using hyperspectral imaging in a high throughput phenotyping greenhouse (Ge et al., 2016; Pandey et al., 2017).

Multivariate analysis techniques are required to relate the spectral information gained from hyperspectral instruments to the chemical or physical traits of interest. Many different multivariate methods exist for model development but these techniques require a measured reference sample to use in the development of calibration models. Although ideal, this is impossible to achieve at a pixel-scale resolution; wet chemistry reference methods often require minimum amounts of tissue from an entire leaf or plant. Therefore, regression is often performed using mean spectra extracted from the entire plant or a region thereof (Ge et al., 2016; Pandey et al., 2017). Regression coefficient vectors based on these mean spectra can then be applied to the image at the individual pixel scale to make predictions at a resolution equivalent to that of the images acquired. These developed prediction maps, or distribution maps, provide a visual interpretation of the content and spatial variation of the predicted component which otherwise cannot be visualized by the hyperspectral data alone.

The present study focused on wheat, the major winter crop in Australia. The specific objectives were (i) to acquire hyperspectral images of wheat plants using a high throughput phenotyping system, (ii) develop regression models capable of predicting water and nitrogen levels of wheat plants, and (iii) apply the regression coefficients from the best-performing models to hyperspectral images in order to develop prediction maps to visualize nitrogen and water distribution within plants.

## Materials and Methods

The experiment was carried out in an automated phenotyping platform (LemnaTec GmbH, Aachen, Germany) at The Plant Accelerator (Australian Plant Phenomics Facility, University of Adelaide, Adelaide, Australia; longitude: 138.64, latitude: -34.97). The platform houses a hyperspectral imaging chamber (WIWAM, Ghent, Netherlands) which contains two individual cameras, a Specim FX10 (Specim, Oulu, Finland) operating in the VNIR (visible and near infrared: 400–1,000 nm) range and Specim SWIR (Specim, Oulu, Finland) operating at the longer SWIR (shortwave infrared: 1,300–2,500 nm) wavelengths. The VNIR FX10 has spatial sampling of 1024 pixels and a spectral interval of approximately 1.3 nm, capturing a total of 448 individual spectral measurements for each image acquisition. The SWIR camera has spatial sampling of 640 pixels and a spectral interval of 5.7 nm, capturing 288 bands. The hyperspectral imaging chamber is illuminated by 18 halogen lights to ensure a consistent light source across the wavelengths.

### Experimental Design

Four soil nutrient factors at two levels each and two watering treatments were applied to four varieties of wheat- cv Gladius, Kukri, Mace and RAC875. The nutrient factors were nitrogen (N:25, 100 mg/kg), phosphorous (P:15, 40 mg/kg), potassium (K:20, 60 mg/kg), and Micromax (5, 10 g/150 g). Half of the full 2^4^(= 16) combinations of the soil nutrient treatments, in addition to a control treatment where no nutrients were added, were included in the design, resulting in nine different soil nutrients treatments (**Table 1**). The base soil was a 1:1:1 mixture of UC (University of California)-mix:coco-peat:clay-loam without any nutrients but balanced to pH 6.4 using dolomite lime. The base soil was divided and different levels of nutrients were added and mixed by hand. Nitrogen was added as Polyon urea (Polyon, 42% N, Koch, Melbourne, Australia), phosphorous as Superphosphate (20.1% P, Incitec Pivot Ltd., Melbourne, Australia), potassium as Potash sulphate (Greenskote, 41% K), and secondary nutrients as Micromax (Scotts Micromax micronutrients; Scotts-Sierra Horticultural Products Co., Marysville, Ohio).

**Table 1:** Nutrient levels of the different soils used in this study. Half of the full 2^4^(= 16) combinations of the soil nutrient treatments were used in addition to a control treatment, where no nutrients were added.

Soil	N (mg/kg)	P (mg/kg)	K (mg/kg)	secondary nutrients (g/150kg)
1	0	0	0	0
2	25	15	20	5
3	100	15	20	10
4	25	40	20	10
5	100	40	20	5
6	25	15	60	10
7	100	15	60	5
8	25	40	60	5
9	100	40	60	10

For the drought treatment, pots were dried down to 10% (g/g) gravimetric water content 23 days after sowing (DAS) to ensure initial plant establishment in well-watered conditions. The well-watered pots were watered to 20% (g/g) water content which was then increased to 23% (g/g) from 34 DAS as growth increased and additional water reserves were required. Each soil, variety and drought combination was replicated six times resulting in a total of 432 pots. Plants were grown in 150 mm pots containing 2.5 kg of dry soil. The greenhouse maintained an average daytime temperature of 23.8°C and average night temperature of 17.5°C.

When first sown, pots were kept on greenhouse benches and hand watered daily until plant emergence. The pots were transferred to the conveyor system 17 DAS where the daily watering became automated. Pots were arranged in a criss-cross design with split-plots, randomized using “dae” (Brien, 2017), a package for the R statistical computing environment (R Core Team, 2017). The pots occupied 24 lanes divided into six zones, each containing a single replicate of allocated factors. The four varieties were randomized to the four lanes in each zone. The layout was split into two sides (west and east) and the drought treatments randomized to the combination of zones by sides using three 2x2 Latin squares. The nine soil nutrient treatments were assigned to the nine carts with a lane-side combination using an 8x9 Youden square.

### Data Collection

#### Hyperspectral System and Data Measurements

Hyperspectral images were collected weekly from 31–61 DAS from a position above the plant. The data collected for each plant consisted of a raw hyperspectral datacube, a white reference and dark reference image. MATLAB (2017b, The MathWorks, Natick, MA) was used to write a function to convert the raw and reference image data into calibrated 3D datacubes of the plants (Equation 1):

(1) $$I_{\mathrm{calibrated}}=\;\frac{I_{\mathrm{raw}}-I_{\mathrm{dark}}}{I_{\mathrm{white}}-I_{\mathrm{dark}}}\;$$

where *I*_calibrated_ is the calibrated datacube image, *I*_raw_ is the raw, unprocessed datacube, *I*_dark_ is the dark current image, and *I*_white_ is the white reference image.

#### Plant Sampling and Chemical Analysis

At the end of the experiment (61 DAS), the flag leaf from the main tiller and the remainder of the plant were harvested separately. The plant (minus a flag leaf) was immediately weighed to measure plant fresh weight. Samples were then placed in an oven at 60°C for 72 hours until a constant weight had been achieved and were then reweighed to obtain dry weight. Plant water content was calculated by (Equation 2):

(2) $$water\;content=\;\frac{W_{F}-W_{D}}{W_{F}}\;x\;100\%$$

where W_F_ is the fresh weight of the harvested sample and W_D_ is the dried sample weight.

The flag leaf samples were reserved for nitrogen analysis. Nitrogen was measured using a “rapid N exceed” N analyzer (Elementar Analysensysteme GmbH, Langenselbold, Germany) with the Dumas combustion method. Samples were dried using the same methods as the whole plant samples and were then ground using a Geno/Grinder (SPEX SamplePrep, NJ, USA). Although different nutrient factors of N, P, and K were selected in the experimental design, only nitrogen values were predicted; there was insufficient flag-leaf tissue to also allow for the analysis of phosphorous and potassium. Nitrogen was therefore selected due to its importance to crop health.

### Data Analysis

#### Extraction of Mean Plant Spectra

In order to extract only the spectral information corresponding to the plant shoot, the background pixels of the images were identified and excluded. This was achieved in the VNIR (400–1,000 nm) images by establishing an enhanced vegetation index (EVI) threshold mask in order to segment the plant tissue from other pixels (Huete et al., 2002). The band at 670 nm was assigned as the red band, 800 nm as the NIR band, and 470 nm as the blue band. These bands were extracted from the datacubes and used to calculate the EVI output for each image (Equation 3):

(3) $$EVI=\frac{2.5\;R_{\mathrm{NIR}}-R_{R}}{(R_{\mathrm{NIR}}+6R_{R}-\;7.5R_{B})+1}$$

where R_NIR_ is the reflectance value in the near infrared band, R_R_ is the reflectance value in the red band, and R_B_ is the reflectance value in the blue band. The EVI output image was found to be very effective at identifying and segmenting plant pixels from background pixels when a threshold of 0.25 was applied. A binary mask based on the EVI values above 0.25 was therefore built and applied to the calibrated images. The reflectance values of all pixels identified as vegetation were then averaged to obtain the average reflectance spectrum of each plant.

For the SWIR images (1,000–2,500 nm), a different segmentation method was adopted (Liu et al., 2019). The SWIR data was first transformed from the original space of the hypercube to a hyper-hue space. A SVM model was then trained with a radial basis function kernel using the svm.OneClassSVM function in the Python programming language (Python Software Foundation) with the sklearn toolbox (Pedregosa et al., 2011) and optimal parameter tuning. The extracted mean spectra from both the VNIR and SWIR cameras were then combined for each plant.

#### Multivariate Regression

The average plant reflectance spectrum extracted from each image was used to develop models capable of predicting water content (%) and nitrogen content (%) in wheat plants. Spectral pre-processing techniques were used to remove noise, transform spectra, emphasis features, and to extract useful information in order to develop multivariate prediction models. The preprocessing techniques selected were adapted from those of Dotto et al. (2018) and Gholizadeh et al. (2015) and included the first-order Savitzky-Golay derivative (see further detail below) on reflectance spectra (SGD1), second-order Savitzky-Golay derivate on reflectance spectra (SGD2), the first-order Savitzky-Golay derivative of the absorbance-transformed spectra (ASGD1), the second-order Savitzky-Golay derivative of the absorbance-transformed spectra (ASGD2), multiplicative scatter correction (MSC), extended multiplicative scatter correction (EMSC), normalization by range (NBR), standard normal variate spectra (SNV), and smoothed spectra (SMO). In addition, the raw spectra were also used as model input to evaluate whether preprocessing actually improved the regression results.

Savitzky-Golay (SG) derivatives are used to reduce baseline shifts and linear trends: the first derivative removes baseline drifts whereas second derivatives can remove both baseline and linear effects (Martens and Næs, 2011). SG derivatives perform least squares linear regression fits of a polynomial around each point in the spectrum to smooth the data. They involve a spectral smoothing method prior to derivation to reduce the signal-to-noise ratio and to determine how many adjacent variables will be used for their calculation. Consideration is required when selecting the tuning parameters for derivatives: the polynomial order, window size, and order of differentiation can strongly influence resulting spectra (Zimmermann and Kohler, 2013). The first-order derivative was calculated using Savitzky-Golay filtering in the “prospectr” package (Stevens and Ramirez-Lopez, 2015) in the R open-source statistical environment (R Core Team, 2007). The SGD1 treatment was fitted with a first order differentiation, second-order polynomial, and a window size of 11. Similarly, the SGD2 treatment was fitted with a second-order polynomial, a window size of 11 but with a second order differentiation. For the ASGD1 treated data, the raw reflectance was first converted to absorbance values before a first-order Savitzky-Golay derivative was applied with a second-order polynomial and a window size of 11 nm.

Multiplicative scatter correction (MSC) involves regressing each spectrum in a dataset against a reference spectrum (quite commonly, the mean spectrum) in order to estimate the intercept and slope of the equation representing the scattering component (Geladi et al., 1985). Each spectrum in the dataset is corrected by subtracting the intercept and dividing by the slope. Extended multiplicative scatter correction (EMSC) is an improvement to MSC that allows the physical light scattering effects to be separated from chemical light effects in spectra (Martens and Stark, 1991).

Standard normal variate (SNV) removes scatter effects from spectral data by performing a row-oriented transformation which centers and scales each spectrum (Barnes et al., 1989). Performing SNV will produce similar results to MSC. The main difference is that SNV does not use the mean spectrum for standardisation but relies only on the data of each individual spectrum. Normalization by range (NBR) is a simple normalisation which adjusts values that are measured on different scales. Neither SNV nor normalization involve least squares fitting and are therefore quite sensitive to spectral noise (Rinnan et al., 2009). The MSC treatment was applied using the “pls” (Mevik and Wehrens, 2007) package in the R open-source statistical environment (R Core Team, 2007). The CRR and SNV treatments were applied using the “prospectr” package (Stevens and Ramirez-Lopez, 2015), the EMSC transformation was applied through the “EMSC” package (Liland, 2017) using a 6-degree polynomial, and the NBR was applied using the “clusterSim” package (Walesiak and Dudek, 2017).

The five different multivariate regression methods applied in this study were partial least square regression (PLSR), Principal Component Regression (PCR), Multiple Linear Regression (MLR), Support Vector Machines (SVM), and Random Forest (RF). PLSR is a multivariate calibration method that uses data compression in order to reduce the full spectrum into a smaller number of noncorrelated components while maintaining the majority of the information contained in the data (Axelsson et al., 2013). PLSR is a popular and widely-used regression method because it performs well when variables contain high correlation or colinearity, as is the case with hyperspectral data, due to its ability to minimise redundancy and overfitting of models (Wold et al., 2001). PLSR was applied in the “pls” package (ncomp = 10, validation = “CV”). The optimal number of components to include in the model was determined by visual inspection of the root mean square error of prediction (RMSEP) graph; the number of components which gave the lowest RMSEP was selected.

PCR is a technique very similar to PLSR in that it is able to model variables when there are a large number of highly correlated predictors present (Wold et al., 1984). Furthermore, both techniques construct new predictor variables which are linear combinations of the original predictor variables. As with the PLSR, PCR was applied in the “pls” package (ncomp = 10, validation = “CV”). The optimal number of components to include in the model was determined by the number of components which gave the lowest RMSEP.

MLR is one of the most common forms of linear regression analysis which has had some success with hyperspectral data (Pan et al., 2016). However, it may not be particularly applicable to all high-dimensional hyperspectral data as many of the data assumptions are not met. For example, MLR assumes that a linear relationship between the independent and dependent variables exists as well as that variables are normally distributed (Montgomery et al., 2012; Osborne and Waters, 2002). The “MASS” package (Venables and Ripley, 2002) was used to fit a linear model by ridge regression (lambda = 0.1-10 by 0.1, repl = 10).

SVM is a group of supervised learning methods originally developed for classification and has recently been adapted for regression models. SVM create hyperplanes that maximize the margins between different classes by reducing the cost function and therefore enabling high prediction performance (Do et al., 2012; Karatzoglou et al., 2005). SVM models are able to fit both linear and nonlinear relationships between variables and are able to handle large datasets (Dotto et al., 2018). Support vector machine (type = “eps”, kernel = “radial”, cost = 50) was employed using the “e1071” package (Meyer et al., 2017).

RF is a machine learning technique that enhances the performance of a single decision tree by averaging the predictions from multiple trees, each of which is generated from a random selection of the input variables (Belgiu and Dragut, 2016). RF is flexible with small or large datasets but their validation performance is usually poor compared to their calibration performance. The results from a RF model can also be difficult to interpret so their implementation has not been widespread in past spectroscopic vegetation studies. The “randomForest” package was used to implement a RF algorithm (ntree = 1000, type = “regression”) (Liaw and Wiener, 2002).

The dataset of mean spectra was split based on experimental replicates into a training set (n = 236 for nitrogen and n = 250 for water) and a validation (n = 109 for nitrogen and n = 105 for water) set to allow both the development and independent validation of the models. Only the training data set was used in the development of the nutrient prediction models; the validation set was used as an independent test set. The total number of samples used in model development deviated from the total number of pots in the experiment (n = 432) due to technical issues during image acquisition. The number of samples in the calibration sets varied between the water and nitrogen regressions because plants grown in soil 1 (no nutrients) did not produce a flag leaf and therefore were not sampled for nitrogen analysis. The accuracy of the developed models was assessed using the independent testing set excluded from the model calibration. Three statistical measures were calculated to evaluate the models developed by each of the multivariate methods: the coefficient of determination (R^2^), root mean square error (RMSE), and the ratio of performance to deviation (RPD) (Equations 4–6). 

(4) $$RMSE=\sqrt{\frac{\sum_{i=1}^{n}{\left({\hat{y}}_{i}-y_{i}\right)}^{2}}{n}}$$

(5) $$R^{2}=\;\frac{\Sigma \;{\left({\hat{y}}_{i}-\bar{y}\right)}^{2}}{\Sigma \;{\left(y_{i}-\bar{y}\right)}^{2}}$$

(6) $$RPD=\frac{SD}{RMSE}$$

where ${\hat{y}}_{i}$ are the predicted values, $\bar{y_{i}}$ is the mean of the observed value, *y* is the observed value, n is the number of samples in the validation or calibration set, and SD is the standard deviation of the reference values.

Since VNIR cameras are more affordable than SWIR cameras, we first tested to see whether models based on VNIR wavelengths alone were able to predict nitrogen and water content in wheat. Full-spectra models incorporating both VNIR and SWIR wavelengths were then trialled to see if models were improved. While adding SWIR data increases the number of variables, it may contribute noise to the original VNIR models rather than adding useful information. SWIR wavelengths alone were not exclusively trialled because of the camera’s coarser spatial resolution. The larger pixel size of the SWIR images would not allow subtle spatial variations to be visualized in the distribution maps.

#### Wavelength Selection

Wavelength selection is widely performed with hyperspectral data because a large number of wavelengths are often redundant and contribute to noise. In this study, wavelength selection was approached in two ways: firstly, using regression coefficients from the full-spectra models and secondly, using only wavelengths located at known-absorption features for both water and nitrogen. For the regression coefficient approach, the vector of regression coefficients (β), a measure of association between each wavelength and the response, was output from the original full-spectra PLSR models. Wavelengths with small absolute regression coefficients, and therefore low association to water or nitrogen, were removed. Both the nitrogen and water datasets were reduced to 132 wavelengths based on the top 30% of regression coefficient values.

A second method, a feature-selection method, was also used for wavelength selection. This is a more traditional approach in which only the wavelengths in a particular region of the spectrum known to be associated with responses (i.e., water or nitrogen content) are selected. Wavelengths known to be related to either water and nitrogen content of vegetation were identified from previous studies (**Tables 2** and **3**). A 40 nm range centered on each of these wavelengths was then included in the models to ensure that the identified wavelengths were encompassed.

**Table 2:** Wavelengths known to be associated with water content in vegetation. A broad range (40 nm) centered on these values were included in the feature-reduced models to ensure that each feature was captured.

Wavelength (nm)	Range Included (nm)	Assignation	Reference
600	580–620	O-H Hydrogen Bonding	Hunt and Rock, 1989
680	660–700	Electron transition	Hong et al., 2017
810	790–830	C-H	Hong et al., 2017
820	800–840	C-H	Hunt and Rock, 1989
860	840–880	C-H	Gao, 1996; Eitel et al., 2006
900	880–920	C-H	Peñuelas et al., 1993; Peñuelas et al., 1995
970	950–990	O-H bend	Curran, 1989; Peñuelas et al., 1993; Peñuelas et al., 1995
1240	1,220–1,260	C-H	Gao, 1996; Eitel et al., 2006
1530	1,510–1,550	N-H secondary amines	Foutry and Baret, 1997; Curran, 1989
1550	1,530–1,570	N-H secondary amines	Musick and Pelletier 1986; Musick and Pelletier, 1988
1720	1,700–1,740	C-H	Foutry and Baret, 1997
1750	1,730–1,770	C-H secondary overtones	Musick and Pelletier 1986; Musick and Pelletier, 1988
2080	2,060–2,100	N-H and C-H, O-H stretch and deformation	Musick and Pelletier 1986; Musick and Pelletier, 1988; Curran, 1989
2350	2,330–2,370	C-H combinations	Musick and Pelletier 1986; Musick and Pelletier, 1988; Curran, 1989
1400-1450	1,400–1,450	O-H bend and stretch	Curran, 1989; De Bei et al., 2011

**Table 3:** From Ecarnot et al. (2013). Wavelengths known to be associated with nitrogen content in vegetation. A broad range (40 nm) centered on these values were included in the feature-reduced models to ensure that each feature was captured.

Wavelength (nm)	Range Included (nm)	Assignation
460	440–480	Electron transition, chlorophyll a,b
530	510–550	Electron transition, carotenoids
670	650–690	Electron transition, chlorophyll a,b
1440	1,420–1,460	O-H bend, first overtone, starch
1500	1,480–1,520	N-H stretch
1680	1,660–1,700	C-H stretch, aromatic
1712	1,692–1,732	C-H stretch, CH3
1770	1,750–1,790	C-H stretch, CH2
1900	1,880–1,920	O-H stretch, C = O, starch, CO2H
1960	1,940–1,980	N-H, CONH2
2080	2,060–2,100	N-H stretch, proteins
2115	2,095–2,135	N-H stretch, CONH2, CONHR
2140	2,120–2,160	Amide, proteins
2230	2,210–2,250	N-H stretch, C = H stretch, amino acid
2300	2,280–2,320	N-H stretch, C = O stretch, amino acid
2400	2,380–2,420	CH2 bend, C-H deformation, cellulose

## Results

**Figure 1:**
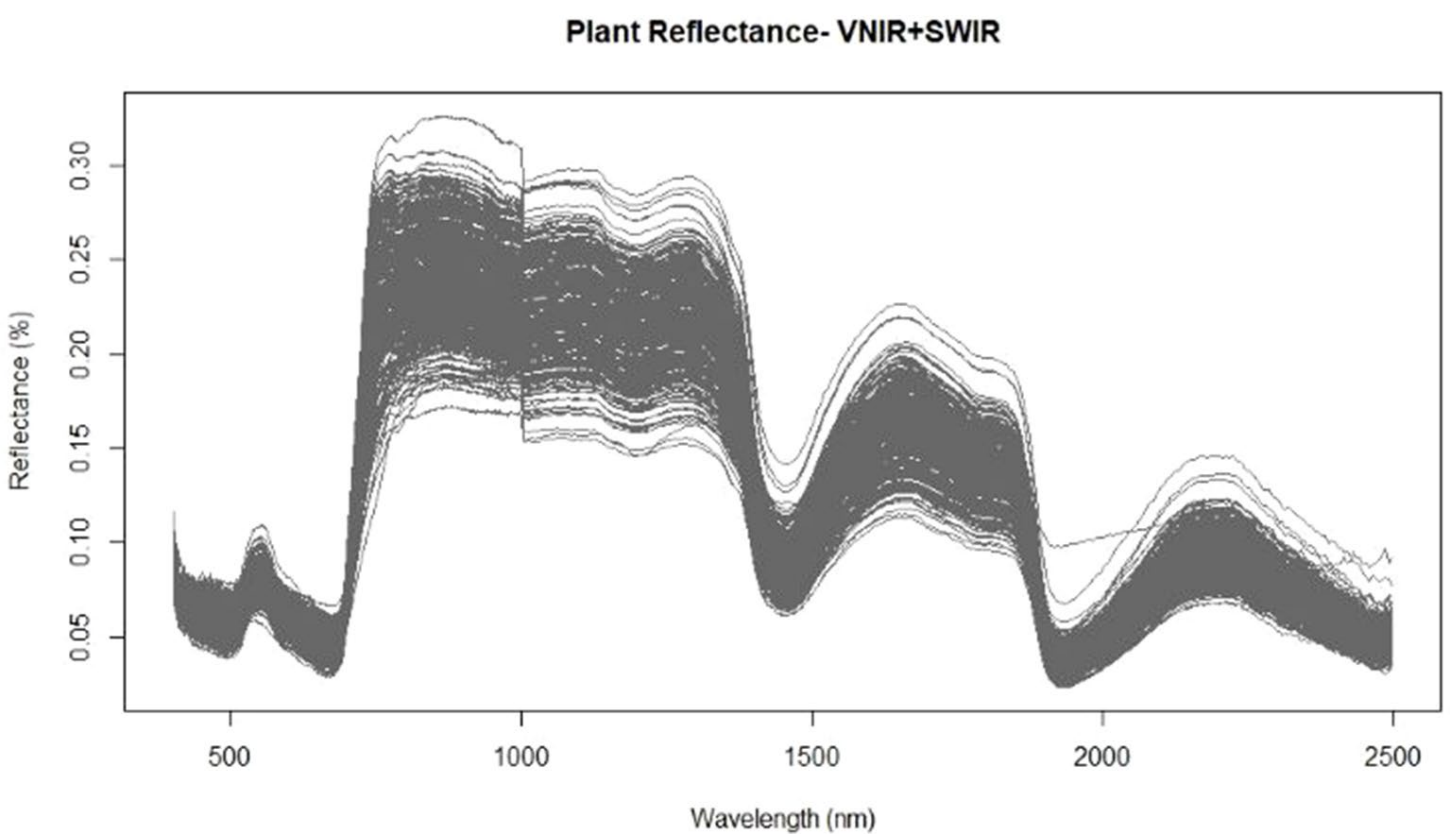
Mean spectra extracted from the vegetation pixels for each image using the FX10 visible and near infrared wavelength (VNIR) camera for wavelengths from 400–1,000nm and the shortwave-infrared wavelength (SWIR) camera for the 1,000–2,500nm wavelengths.

Prior to destructive harvest of the wheat plants grown in varying nutrient and water regimes, hyperspectral images were acquired in the VNIR and SWIR regions. For each corrected hyperspectral image, the pixels corresponding to vegetation were identified and the mean reflectance spectra of those pixels were calculated to develop prediction models for both water content and nitrogen levels. The mean spectra exhibited the typical reflectance properties of vegetation with low reflectance across visible wavelengths, a dramatic increase in reflectance at the transition from visible to near infrared wavelengths, and maximum reflectance values throughout the near infrared domain (**Figure 1**). An obvious feature of the spectral graph was the offset at 1,000 nm. This was caused by the two separate cameras: the FX10 operating at 400–1,000 nm and the SWIR camera operating at 1,000–2,500 nm. The two cameras, while focally and geometrically aligned, had different optical systems and spectral and spatial resolutions, which attribute to the offset between cameras. While overlapping regions common to both cameras were removed (996–1,006 nm), appropriate preprocessing should be applied in order to correct the spectral jump in future studies. Baseline shifts across the spectra were also apparent which suggests that a preprocessing technique which is able to correct for additive scatter, such as EMSC or MSC, may improve subsequent regression results.

### VNIR Wavelengths Alone Are Not Sufficient to Predict Water Content

Initially, only VNIR (400–1,000 nm) wavelengths were used as input into the regression models. The different multivariate regression techniques and preprocessing methods resulted in various predictive performances for quantifying water content. Only three preprocessing methods (ASGD1, SMO, and raw) achieved R^2^ values above 0.50 (**Table 4**). Of the preprocessing methods, the smoothed and raw data consistently achieved the highest overall prediction performance with validation R^2^ values of up to 0.62 and 0.57 for smoothed and raw data, respectively (when used in combination with multiple linear regression). For the regression methods, PLSR models produced the highest accuracy performance for the prediction of water content in fresh wheat leaves; they consistently returned models with higher R^2^ values compared to other techniques with relatively high RPD values and low RMSE values. PLSR validation R^2^ values varied from 0.33 to 0.56, RMSE varied from 3.61–2.85, and RPD varied from 1.18–1.5 (**Supplementary Table 1**). PLSR achieved the best results when used in conjunction with raw spectra, achieving validation statistics of R^2^ = 0.56, RMSE = 2.85, and RPD = 1.50. However, even this model does not provide a sufficient level of accuracy for predicting water content. According to the model classification of Li et al. (2018), these models can only be considered as “acceptable” for the prediction of water content based on their R^2^ and RPD values (0.50≤R^2^ ≤ 0.75 and 1.40≤RPD ≤ 2.00).

**Table 4:** Performance results for the prediction of water content in wheat using VNIR (400–1,000 nm) spectra.

Calibration						
		PLSR	PCR	MLR	RF	SVM
ASGD1	R^2^	0.63	0.42	0.50	0.47	0.84
	RMSE	2.92	3.61	3.42	3.46	1.96
	RPD	1.63	1.32	1.39	1.38	2.43
SMO	R^2^	0.64	0.60	0.61	0.44	0.67
	RMSE	2.90	3.04	2.96	3.57	2.76
	RPD	1.64	1.57	1.61	1.33	1.73
Raw	R^2^	0.67	0.60	0.57	0.44	0.69
	RMSE	2.79	3.04	3.13	3.55	2.70
	RPD	1.71	1.56	1.52	1.34	1.77
Validation						
		PLSR	PCR	MLR	RF	SVM
ASGD1	R^2^	0.51	0.41	0.54	0.49	0.52
	RMSE	3.01	3.30	2.94	3.06	2.96
	RPD	1.42	1.29	1.45	1.39	1.44
SMO	R^2^	0.56	0.54	0.62	0.47	0.55
	RMSE	2.90	2.88	2.70	3.11	2.89
	RPD	1.47	1.48	1.58	1.37	1.48
Raw	R^2^	0.56	0.54	0.57	0.49	0.56
	RMSE	2.85	2.88	2.82	3.05	2.86
	RPD	1.50	1.48	1.51	1.40	1.49

R^2^, coefficient of determination, RMSE, root mean square error; RPD, ratio of performance to deviation. PLSR, partial least-squares regression; PCR, principal components regression; MLR, multiple linear regression; RF, random forest; SVM, support vector machine; SMO = smoothed.

### VNIR Wavelengths Are Not Sufficient for Nitrogen Prediction

As with water prediction, PLSR and MLR showed the strongest prediction of nitrogen (**Table 5**). The calibration R^2^ values ranged from 0.23 to 0.90 with the calibration RMSE and RPD values varying from 0.55 to 0.22 and 1.13 to 2.84, respectively. On the other hand, the validation R^2^ values ranged from 0.06 to 0.59 with the validation RMSE and RPD values varying from 0.97 to 0.41 and 0.66 to 1.56, respectively (**Supplementary Table 2**). As with the water models, the smoothed and raw data achieved the highest overall prediction performance for estimating nitrogen, considering both calibration and validation data. PLSR models also produced the highest accuracy performance for nitrogen prediction compared to other multivariate methods trialled. For different preprocessing methods, validation R^2^ values varied from 0.26 to 0.59, RMSE varied from 0.55–0.41 and RPD varied from 1.16–1.56 (**Supplementary Table 2**). PLSR achieved the best results when used in conjunction with smoothed spectra, achieving validation statistics of R^2^ = 0.59, RMSE = 0.41, and RPD = 1.56 (**Table 5**). However, no model developed with VNIR wavelengths alone can be considered as accurate for predicting nitrogen content. As with the VNIR water content models, these regressions are only considered as “acceptable” (0.50≤R^2^ ≤ 0.75 and 1.40≤RPD ≤ 2.00) (Li et al., 2018).

**Table 5:** Performance results (validation R^2^≥0.5) of trialled preprocessing and multivariate methods for the prediction of nitrogen in wheat.

Calibration						
		PLSR	PCR	MLR	RF	SVM
SMO	R^2^	0.56	0.50	0.53	0.42	0.60
	RMSE	0.42	0.45	0.43	0.48	0.40
	RPD	1.49	1.40	1.46	1.31	1.58
Raw	R^2^	0.59	0.49	0.48	0.42	0.62
	RMSE	0.41	0.45	0.45	0.47	0.39
	RPD	1.53	1.38	1.39	1.32	1.60
**Validation**						
		PLSR	PCR	MLR	RF	SVM
SMO	R^2^	0.59	0.54	0.57	0.33	0.43
	RMSE	0.41	0.44	0.42	0.52	0.48
	RPD	1.56	1.47	1.53	1.22	1.33
Raw	R^2^	0.57	0.52	0.58	0.33	0.43
	RMSE	0.42	0.44	0.42	0.52	0.48
	RPD	1.53	1.44	1.54	1.23	1.33

R^2^ = coefficient of determination, RMSE; root mean square error; RPD; ratio of performance to deviation. PLSR; partial least-squares regression; PCR; principal components regression; MLR; multiple linear regression; RF; random forest; SVM; support vector machine; SMO; smoothed.

### Full-Spectra (VNIR+SWIR) Regressions Improve Accuracies for Water and Nitrogen

Since both water and nitrogen also express strongly in the SWIR region, SWIR wavelengths were also included in the regression models to see whether prediction accuracies were improved compared to VNIR models alone. The incorporation of the SWIR wavelengths in the full-spectra models improved the prediction accuracies of both the water and nitrogen models (**Table 6**). For the prediction of water, the validation R^2^ values ranged from 0.34 to 0.63 with the validation RMSE and RPD values varying from 5.22 to 2.8 and 0.88 to 1.64, respectively. Similarly, for the prediction of nitrogen, the validation R^2^ values ranged from 0.14 to 0.66 with the validation RMSE and RPD values varying from 0.63 to 0.41 and 1.04 to 1.64, respectively. As was found with the VNIR models, the smoothed and raw data had the highest overall prediction performance for both water and nitrogen, except for the RF and SVM prediction of nitrogen which was improved with preprocessing. PLSR models again had the most consistently high performance compared to other multivariate methods trialled, particularly when used with raw or smoothed data (**Table 6**).

**Table 6:** Validation prediction accuracies for full-spectra (VNIR+SWIR: 400-2500nm) regression models for predicting water content and nitrogen.

Water full-spectra (VNIR+SWIR) validation						
		PLSR	PCR	MLR	RF	SVM
ASGD1	R^2^	0.56	0.59	0.56	**0.60**	**0.61**
	RMSE	3.11	2.95	3.25	**2.92**	**2.88**
	RPD	1.48	1.56	1.41	**1.58**	**1.60**
ASGD2	R^2^	0.58	0.50	0.34	0.59	0.55
	RMSE	3.07	3.24	5.22	3.00	3.10
	RPD	1.50	1.42	0.88	1.53	1.48
EMSC	R^2^	0.57	0.59	0.54	0.55	0.59
	RMSE	3.10	3.01	3.27	3.08	2.94
	RPD	1.49	1.53	1.41	1.49	1.57
MSC	R^2^	0.57	0.56	0.54	0.49	0.55
	RMSE	3.09	3.10	3.19	3.37	3.09
	RPD	1.49	1.49	1.44	1.37	1.49
SGD1	R^2^	0.58	0.58	0.54	**0.60**	0.59
	RMSE	3.01	3.00	3.25	**2.92**	2.94
	RPD	1.53	1.54	1.42	**1.58**	1.57
SGD2	R^2^	0.52	0.57	0.37	0.57	0.58
	RMSE	3.19	3.03	4.42	3.07	3.00
	RPD	1.44	1.52	1.04	1.50	1.54
SNV	R^2^	0.57	0.57	0.54	0.48	0.55
	RMSE	3.10	3.08	3.19	3.37	3.10
	RPD	1.48	1.49	1.44	1.37	1.48
SMO	R^2^	**0.63**	**0.62**	**0.61**	**0.61**	0.59
	RMSE	**2.80**	**2.83**	**2.96**	**2.89**	2.97
	RPD	**1.64**	**1.62**	**1.56**	**1.59**	1.55
Raw	R^2^	**0.63**	**0.62**	0.58	**0.62**	**0.60**
	RMSE	**2.81**	**2.83**	3.01	**2.85**	**2.95**
	RPD	**1.64**	**1.62**	1.53	**1.61**	**1.56**
**Nitrogen full-spectra (VNIR+SWIR) validation**						
		PLSR	PCR	MLR	RF	SVM
ASGD1	R^2^	0.58	0.57	0.55	0.56	0.55
	RMSE	0.45	0.49	0.46	0.45	0.47
	RPD	1.46	1.36	1.43	1.46	1.42
ASGD2	R^2^	0.51	0.14	0.29	0.54	0.59
	RMSE	0.48	0.63	0.63	0.47	0.45
	RPD	1.39	1.06	1.04	1.41	1.48
EMSC	R^2^	0.56	**0.61**	0.54	0.48	0.53
	RMSE	0.45	**0.46**	0.46	0.49	0.47
	RPD	1.47	**1.44**	1.45	1.36	1.40
MSC	R^2^	**0.61**	0.53	0.54	0.39	0.43
	RMSE	**0.43**	0.49	0.45	0.52	0.52
	RPD	**1.54**	1.35	1.48	1.26	1.28
SGD1	R^2^	0.57	0.58	0.55	0.48	0.55
	RMSE	0.46	0.47	0.45	0.52	0.47
	RPD	1.45	1.41	1.46	1.27	1.40
SGD2	R^2^	0.48	0.43	0.26	0.51	0.56
	RMSE	0.48	0.51	0.63	0.49	0.45
	RPD	1.38	1.31	1.05	1.36	1.46
SNV	R^2^	0.57	0.50	0.55	0.36	0.42
	RMSE	0.45	0.51	0.45	0.54	0.52
	RPD	1.46	1.30	1.48	1.23	1.27
SMO	R^2^	**0.66**	**0.63**	**0.63**	0.37	0.43
	RMSE	**0.41**	**0.44**	**0.41**	0.52	0.50
	RPD	**1.61**	**1.52**	**1.64**	1.26	1.32
Raw	R^2^	**0.60**	**0.61**	0.59	0.36	0.43
	RMSE	**0.43**	**0.44**	0.43	0.53	0.50
	RPD	**1.54**	**1.52**	1.56	1.25	1.33

R^2^ = coefficient of determination; RMSE, root mean square error; RPD, ratio of performance to deviation. PLSR, partial least-squares regression; PCR, principal components regression; MLR, multiple linear regression; RF, random forest; SVM, support vector machine; ASGD1, absorbance transformation then Savitzky-Golay first derivative; ASGD2, absorbance transformation then Savitzky-Golay second derivative; EMSC, extended multiplicative scatter-correction; MSC, multiplicative scatter-correction; SGD1, Savitzky-Golay first derivative; SGD2, Savitzky-Golay second derivative; SNV, standard normal variate; SMO, smoothed. Models with validation R^2^≥0.6 are in bold.

### Wavelength Selection Using Regression Coefficients Reduces Data Without Compromising Accuracy

Wavelength selection was performed in order to remove some of the redundant information inherent in hyperspectral data. Two approaches to wavelength selection were used: through the regression coefficients from the full-spectra models and by selecting only wavelengths located at known-absorption features for both water and nitrogen.

The wavelengths retained in the reduced β coefficient water model were 398–404, 410–415, 426–432, 440–443, 637–705, 724–752, and 950–1,000 nm. Direct associations between reflectance spectra and water content are those features related specifically to the water content in the leaves. These are known to occur at 1450 and 1940 nm with smaller features at 980, 1150, and 1400 nm associated with the bonding and stretching of O-H molecules (Curran, 1989; Ollinger, 2011). However, reflectance properties can also be influenced by indirect effects, those associated with other traits that vary alongside water or nitrogen status. These indirect effects can be caused by properties such as varying leaf pigments or vegetation architecture, which are represented by reflectance changes throughout visible and near-infrared wavelengths (Ollinger, 2011). The fact that the model produced predictions based on wavelengths within only the visible and near-infrared regions suggests that rather than directly quantifying water content, it is detecting indirect associations between water and secondary traits influencing the overall reflectance.

The nitrogen wavelengths with the highest β coefficients were located at 510–637 nm and 693–739 nm. The 693–739 nm region is likely representing changes in the red-edge position, which have previously been shown to vary with crop chlorophyll concentration (Horler et al., 1983; Curran et al., 1990). However, the identified wavelengths from 510–637 nm do not correspond to a direct association with nitrogen. Foliar nitrogen is predominantly found within proteins, largely chlorophyll or Rubisco (Evans, 1989; Elvidge, 1990). Protein absorption features only occur in the infrared region around 910, 1,020, 1,510, 1,690, 1,980, 2,060, 2,130, 2,180, 2,240, and 2,300 nm while chlorophyll absorptions are found in the visible region of the spectrum near 430, 460, 640, and 660 nm (Curran, 1989). Therefore, the 510–637 nm wavelengths are likely detecting an indirect association between the spectra and nitrogen content.

The second approach to wavelength reduction was to use only wavelengths that have previously been related to either water and nitrogen content. The wavelengths identified by previous studies included both direct and indirect associations to water (**Table 2**) and nitrogen (**Table 3**). The wavelengths selected for the reduced nitrogen models excluded some of the direct spectral asssociations known to exist with nitrogen. Wavelengths with direct association to nitrogen occurring within the SWIR region were not included in the model because those absorption features are generally weaker (Curran, 1989; Ollinger, 2011). Therefore, wavelengths were selected (from Ecarnot et al., 2013) which are known to be correlated with nitrogen content in vegetation, even if they are detecting secondary traits relating to changes in water content. Similarly, some of the major water absorption features, those located at 1,940 and 1,150 nm were excluded from the reduced water models while other wavelengths that don’t have direct associations to water were included (e.g., those located within the visible region). A number of plant functions are related to changes in water content. Therefore, even if the exact compound or process is not determined, spectral features of several secondary traits and processes have been used, and included in these reduced water models, for indirect associations to water content.

**Figure 2:**
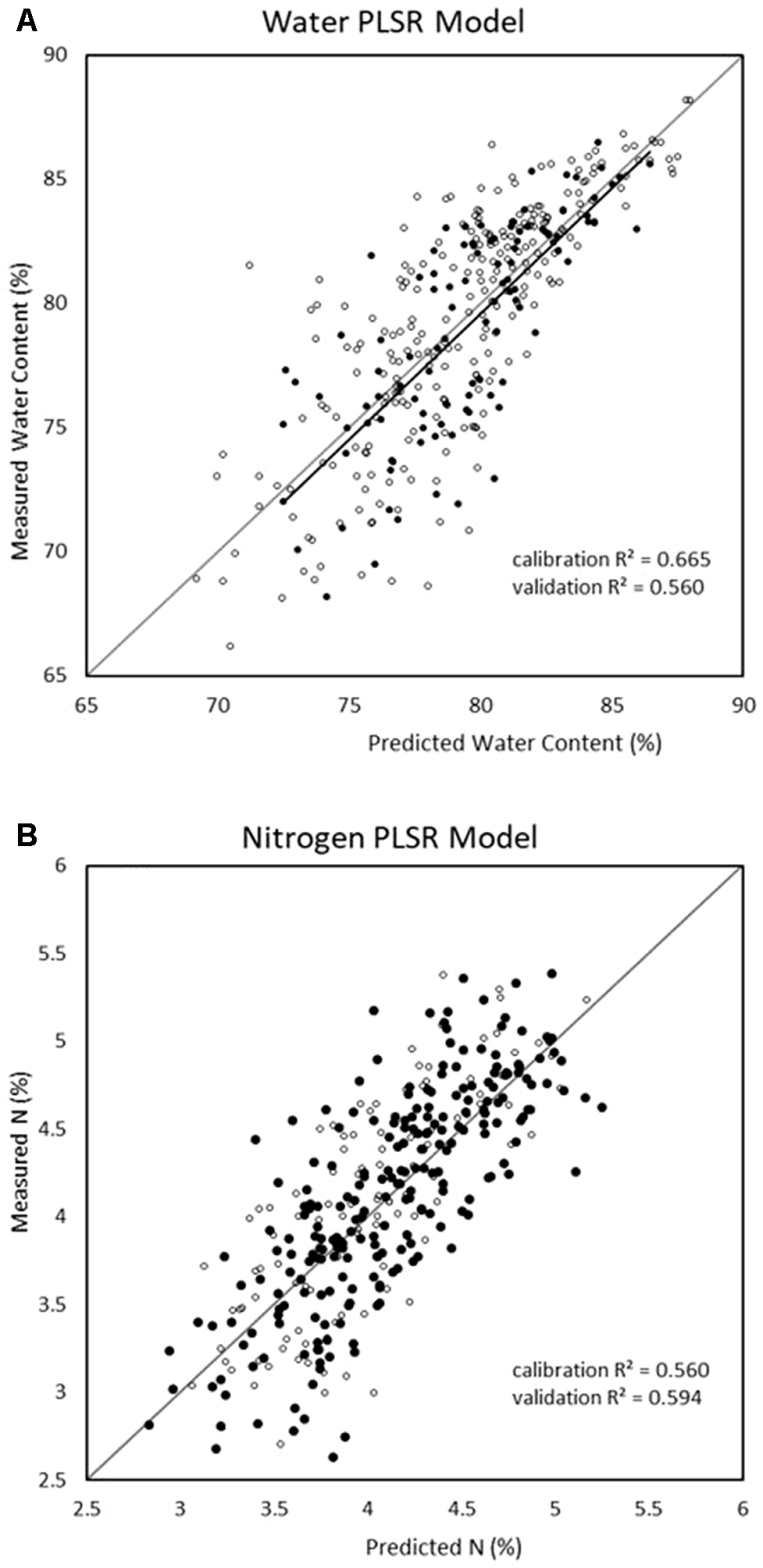
Water (top) and nitrogen (bottom) regression graphs for the models showing the best validation predictive performance from all trialled methods (regression coefficient reduction method with raw spectra and partial least square regression (PLSR)).

Since PLSR consistently showed the strongest performance in the previous model development, it was the only multivariate technique considered in the reduced-wavelength models. The predictive performances of the wavelength-selection models developed using the regression coefficient method had higher accuracy than the full-spectra or VNIR model (**Figure 2**). For the prediction of water content, the regression coefficient method generated a PLSR model with validation R^2^ = 0.69, RMSE = 2.53, and RPD = 1.78 while the feature method gave accuracies of R^2^ = 0.64, RMSE = 2.74, and RPD = 1.76. (**Table 7**). This is compared to a maximum validation R^2^ value of 0.63 using the full-spectra PLSR model (**Table 8**). For nitrogen, the regression coefficient method had a validation accuracy of R^2^ = 0.66, RMSE = 0.41, and RPD = 1.66 (**Table 7**), compared to a validation R^2^ value of 0.60 using the same methods (PLSR with raw data) from the full-spectra models (**Table 8**).

**Table 7:** Predictive performances for the wavelength refined models. Cal = calibration. Val = validation. Results are shown for the two different approaches used for variable selection: using the top 30% of wavelengths based on the regression coefficients from the full-spectra models and using wavelengths previously determined to be associated with nitrogen and water.

Water wavelength selection models				Nitrogen wavelength selection models			
	PLSR	Cal	Val		PLSR	Cal	Val
Regression Coefficients	R^2^	0.81	0.69	Regression Coefficients	R^2^	0.74	0.66
	RMSE	2.05	2.53		RMSE	0.32	0.41
	RPD	2.31	1.78		RPD	1.89	1.66
Known Absorption Features	R^2^	0.71	0.64	Known Absorption Features	R^2^	0.54	0.52
	RMSE	2.57	2.74		RMSE	0.42	0.47
	RPD	1.85	1.76		RPD	1.45	1.39

**Table 8:** Validation results for the strongest performing models for water and nitrogen prediction across the different pre-processing and regression methods trialled- PLSR in combination with raw input data. Wavelength selection using the regression coefficient method produced the strongest models.

		VNIR	Full spectra (VNIR+SWIR)	Wavelength selection regression coefficients	Wavelength selection feature positions
**Water**	R^2^	0.56	0.63	0.69	0.64
	RMSE	2.85	2.81	2.53	2.74
	RPD	1.50	1.64	1.78	1.76
**Nitrogen**	R^2^	0.57	0.60	0.66	0.52
	RMSE	0.42	0.43	0.41	0.47
	RPD	1.53	1.54	1.66	1.39

The reduced wavelength models, while greatly simplifying the models also reduced the degree of overfitting. The calibration and validation prediction values (**Table 7**) are much closer together than the models without wavelength selection (**Tables 4** and **5**). The feature method produced accuracies of R^2^ = 0.52, RMSE = 0.47, and RPD = 1.39 for predicting nitrogen. The feature reduction method for wavelength refinement did not improve either the water or nitrogen models.

### Distribution Maps Can Visualize Water and Nitrogen Distribution

**Figure 3:**
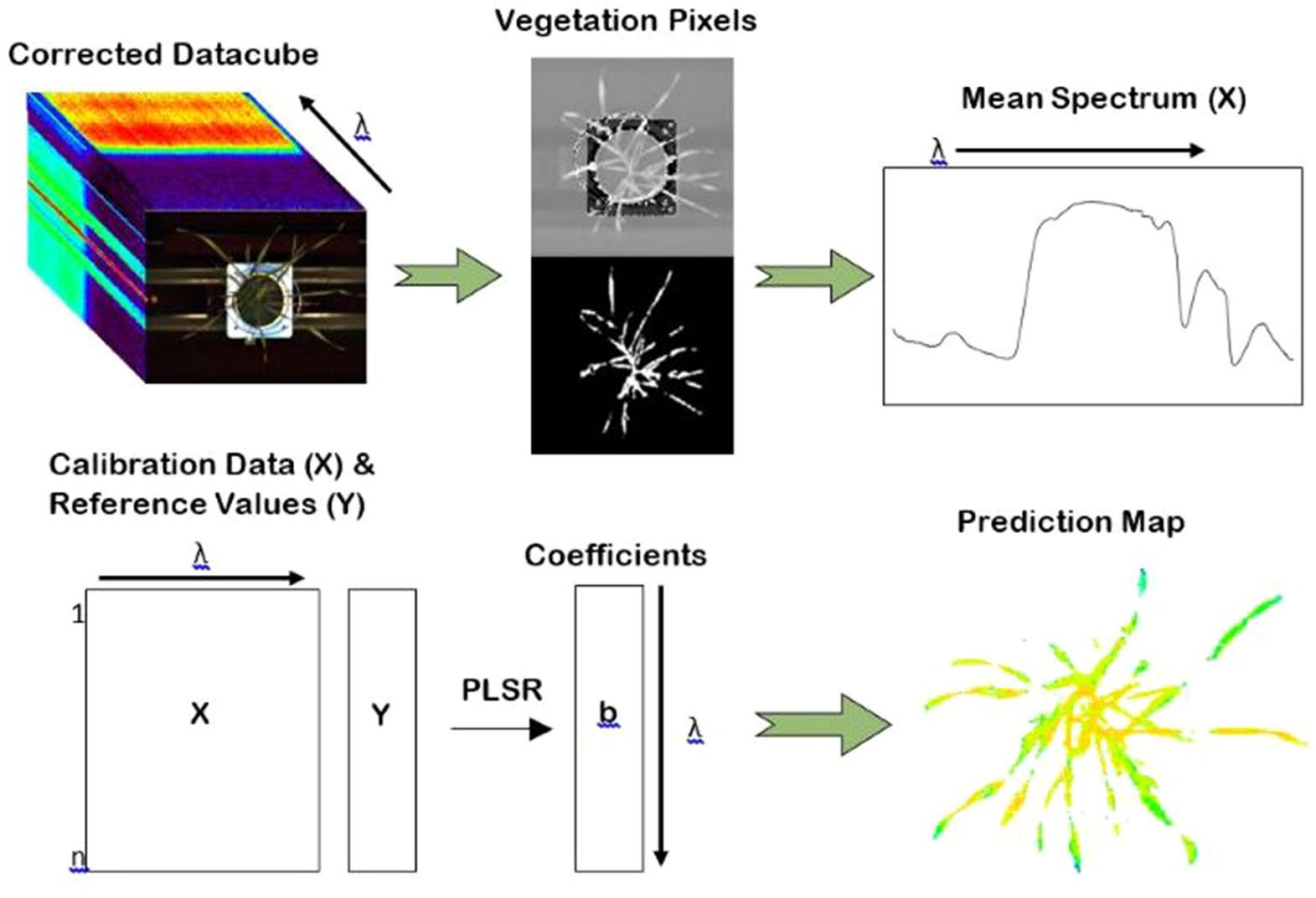
The procedure for the development of water and nitrogen distribution maps. Mean spectra were used as input to generate a PLSR model. The coefficients of the partial least square regression (PLSR) model were then applied to the unfolded datacube at the individual pixel level providing a spatial visualisation of water and nitrogen distribution within the plants.

Distribution maps, or prediction maps, were developed in order to provide a visual representation of the concentration of water and nitrogen content within the plants as well as to show any spatial variability within individual plants. Distribution maps were created by applying the regression coefficients of the highest performing VNIR models to the calibrated hyperspectral images (**Figure 3**). In the case of water content, the coefficients from the VNIR PLSR model of raw spectra were used. For nitrogen, the coefficients from the VNIR PLSR model with smoothed spectra were applied. Only the VNIR models were used to develop the distribution maps; the combined full-spectra models were not considered. This is because the two cameras (FX10 and SWIR) operate at different spatial resolutions, so the combined models cannot be visualized on a single image.

**Figure 4:**
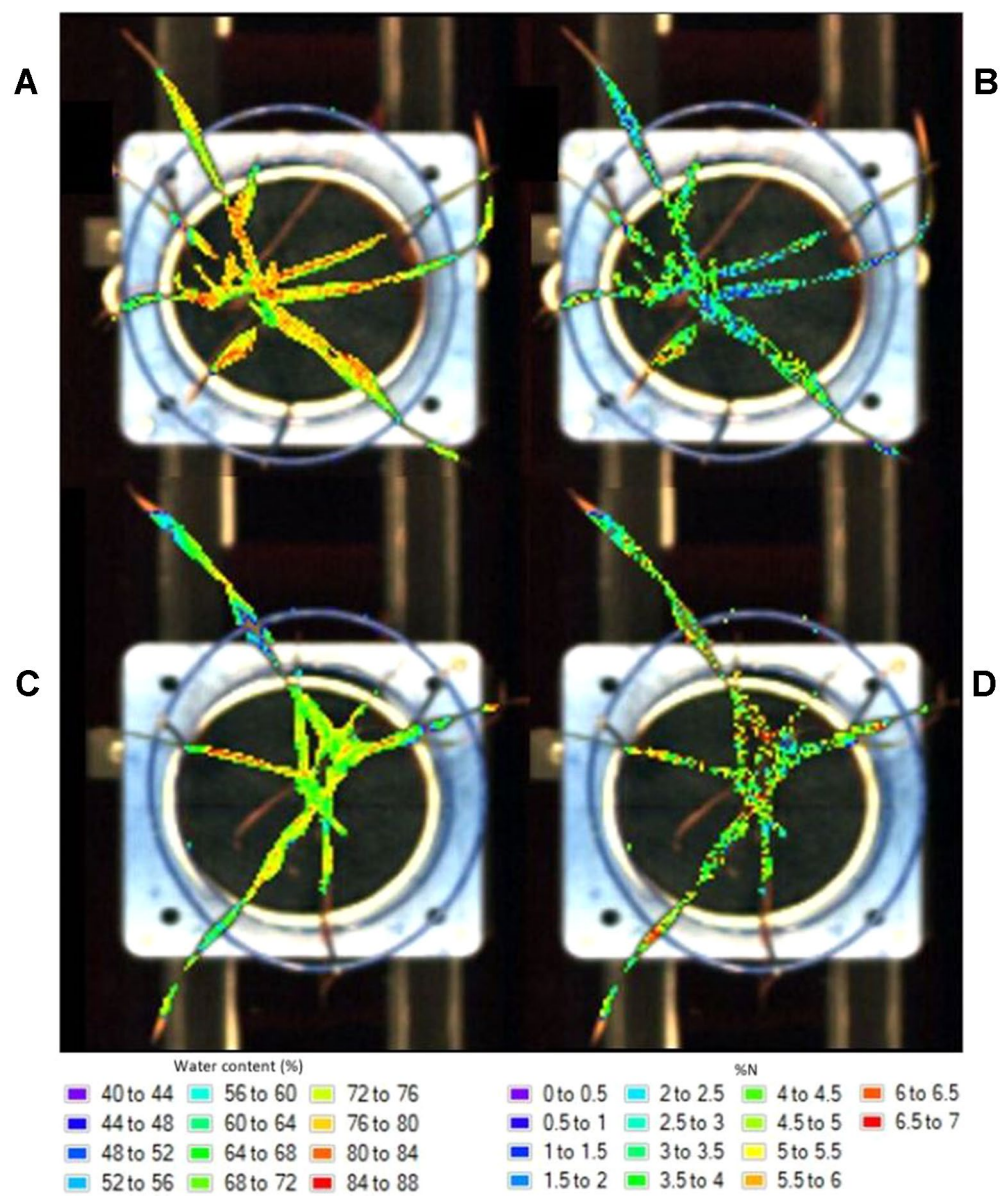
Distribution maps showing the prediction of water content in a watered **(A)** and drought **(C)** plant and nitrogen levels in a low **(B)** and high **(D)** nitrogen soil plant.

The resulting prediction maps revealed the spatial variation in biochemical properties, particularly water content, and allowed for a visual comparison between and within the plants which is otherwise impossible with the raw hyperspectral data (**Figure 4**). There were noticeable differences between the maps of the watered (**Figure 4A**) and drought (**Figure 4C**) plants as indicated by their color scale; the watered plants appear yellow-red (72–88% water content) whereas the drought plants are predominantly green (64–72% water content). This is also the case for the low (**Figure 4B**) and high (**Figure 4D**) nitrogen plants, however this difference is less obvious.

In general, water content was higher at the base of the leaves and decreased toward the tips (**Figures 4A, C**). Higher levels of water were also apparent around the midrib region as opposed to the outsides of the leaves. These clear patterns and gradual degradation between neighboring pixels suggests that the water distribution maps provide a visually plausible indication of spatial variability within the plants. The nitrogen distribution maps (**Figures 4B, D**) do not appear to follow a plausible spatial pattern; each pixel is a different colour with no clear gradation to the neighboring pixels. This “noise” in the image is likely the result of model overfitting (Gowen et al., 2014).

## Discussion

Hyperspectral imagery combines the spatial information of traditional RGB imagery with the benefits of high-resolution spectral reflectance data. This reflectance data, an indication of how light is interacting with the target, provides a unique spectral fingerprint of the chemical nature of each plant. However, hyperspectral images alone are not visually intuitive or easily interpretable. The development of distribution maps has allowed for a visual comparison between and within plants and revealed the spatial variation in water and nitrogen.

Of the five different multivariate regression methods and 10 different spectral preprocessing techniques trialled, the smoothed and raw data consistently achieved the highest overall prediction performances for both water and nitrogen. This suggests that preprocessing may not have been required for this dataset and that applying preprocessing techniques may have dampened some of the important features of the spectra. Without accounting for the physical influence of scatter or noise, it would normally be expected that a poorer performance is achieved for the smoothed data in comparison to pre-processed data, however, this was found not to be the case.

The high accuracies achieved with PLSR in comparison to other techniques is due to its ability to handle both the high dimensionality and collinearity inherent in hyperspectral data and its effectiveness when a large number of collinear predictor variables are present (Wold et al., 1984). The strong performance of PLSR and its ability to handle spectral data explains why it has been so widely adopted in past hyperspectral studies quantifying plant biochemical properties. MLR and SVM also performed well.

One apparent and perhaps significant finding was the large difference between the calibration and validation performances for the VNIR models (**Supplementary Tables 1** and **2**). The inconsistencies between the sets, particularly in the case where validation statistics are considerably lower than calibration statistics, may be attributed to model overfitting. This discrepancy is not as severe in the wavelength-selection models suggesting that the wavelength selection methods were able to remove the noise and irrelevant wavelengths in the data. The prediction accuracies between the nitrogen and water models were quite similar; there was no drastic difference in the ability to predict one property over the other. This is true for all models developed: VNIR, full-spectra, and wavelength-refined models (besides perhaps the absorption feature method for wavelength-selection which yielded validation R^2^ = 0.64 for water and R^2^ = 0.52 for nitrogen).

The stronger performing models, e.g., PLSR, were not drastically improved in the full-spectra models in comparison to the VNIR models. However, the regression and preprocessing methods that did not perform as well in the VNIR models, e.g., EMSC and multiple linear regression, were improved significantly. By considering only the visible and near infrared wavelengths initially, it might have been expected that nitrogen and water, both which have major spectral expressions in the shortwave infrared region, would not be predicted accurately. The fact that the full-spectra (VNIR+SWIR) models did not significantly improve the VNIR models suggests that perhaps neither water nor nitrogen content is being directly detected, rather secondary processes within the plant spectrally expressed in the VNIR region.

The applied wavelength reduction techniques, both the “regression coefficient” and “known absorption feature” methods, while successfully reducing the volume of data, did not significantly improve the model accuracies. The wavelengths incorporated in the two approaches contained both direct and indirect associations with nitrogen and water. Since the regression coefficient approach is likely detecting both direct and indirect associations, selecting known absorption features also based on both direct and indirect associations does not necessarily provide additional or new information (i.e., there is some degree of duplication in the two approaches since they both target direct and indirect associations). If only direct features of nitrogen or water were selected from previous literature during the known absorption feature approach, then the wavelength selection methods could be considered as more independent approaches; one approach, the known absorption feature approach, is targeting only the direct associations to water or nitrogen while the regression coefficient approach is targeting both direct and indirect associations. Targeting only direct associations would mean that the developed models would likely have a stronger predictive performance for independent datasets. Due to the indirect associations included, the wavelengths identified as being sensitive to water and nitrogen cannot be generalised and may not hold true for all future studies.

Previous studies have utilized high-throughput phenotyping platforms equipped with hyperspectral cameras for investigating crop traits. Pandey et al. (2017) imaged maize and soybean leaves using a hyperspectral camera with a spectral range of 550–1700 nm to predict leaf water content and nutritional status. They showed that both water and nitrogen content could be predicted accurately using PLSR (R^2^ = 0.93 and 0.92 with RPD = 3.80 and 3.60, for water and nitrogen respectively). Ge et al. (2016) used high throughput hyperspectral imaging to characterize the temporal dynamics of the leaf water content of maize. They used PLSR to accurately predict leaf water content using a hyperspectral camera with a wavelength range of 550–1750 nm and a spectral bandwidth of 5 nm (R^2^ = 0.87, RMSE = 3.0%, and RPD = 2.82). Although different species and smaller datasets were used (Ge et al. (2016) used 80 plants while Pandey et al. (2017) used 120), these previous studies achieved much greater accuracies for predicting water and nitrogen content than presented here. Compared to wheat, which has narrow, twisting leaves with irregular structure, maize has considerably broader leaves with a more regular leaf architecture. There are fewer pixels to work with when analyzing wheat plants with a greater proportion suffering from mixed signals (i.e., edge pixels of background and plant material). The fact that maize plants have more pixels to work with, and proportionally fewer mixed-pixels, may have contributed to the higher accuracies achieved in these previous studies.

The generally poor R^2^ values achieved in this study can be attributed to a number of factors. The main factor influencing the poor performance of the nitrogen models is likely the different scales at which the spectral and reference measurements were made. The spectra were extracted and averaged over the entire plant whereas the reference laboratory measurements of nitrogen were taken on a single leaf. Nitrogen is considered a mobile element within plants and can be retranslocated to younger leaves from older leaves (Taiz and Zeiger, 1998). Its distribution is not homogenous throughout wheat plants but is generally lower in older leaves (Wang et al., 2005). Therefore, the nitrogen measured in a single leaf may not be representative of the entire plant’s nitrogen status. Regressions may be improved if either spectra were extracted from only the leaf which was sampled for nitrogen (i.e., the flag leaf), or alternatively, reference nitrogen measurements were determined on the plant scale. This could be achieved by calculating masks which identify only the flag leaf within the image. However, the flag leaf could not be identified automatically in the images. Manually extracting the flag leaf would have been too time-consuming and would have diminished the benefits of using a high-throughput system. By separating the flag leaf from the remainder of the plant material and using only the leaf pixels corresponding to those analysed for nitrogen, regression results are likely to improve. Similarly, water regressions could also be improved by reversing the mask and removing the flag leaves which were not used in the calculation of water content. A smaller subset of the data could be used to this affect to assess how much of the noise and error can be attributed to different measurement scales.

Alternatively, the poor results for the water regressions may be attributed to measurement protocols used to obtain reference values. The relative water content (RWC), or relative turgidity of a leaf or plant, is a measure of its hydration status (actual water content) relative to its maximal water holding capacity at full turgidity (Weatherley, 1950; Mullan and Pietragalla, 2012). Determining the RWC may have provided a better indication of plant water status than the method used here which did not consider turgid weight. Alternatively, regressions may have been stronger if leaf thickness was also considered. Light reflected from leaves or transmitted through leaves depends on both the RWC of leaf cells as well as the thickness of the leaves (Seelig et al., 2008). Therefore, models may have proved more accurate if leaf thickness or even total plant biomass were taken into consideration.

A further contribution to the poor R^2^ values for both the water and nitrogen regressions may be the canopy geometry. The geometry of plants and inclination of individual leaves has a strong influence on the spectral information acquired (Behmann et al., 2015; Huang et al., 2018). The cameras took a top-view image from directly above the plant. Lower leaves may have been hidden by leaves above and this information consequently missed. Incorporating 3-D structural information alongside the hyperspectral images may reduce the effect of plant geometry on the acquired spectra.

Prediction accuracies may also improve if the spectral reflectance jump between the two sensors, caused by the different properties and measurement principles of the cameras and detectors, was corrected. Overlapping wavelength regions (996–1006 nm) were removed from the spectra, however, correcting the spectral jumps with appropriate preprocessing techniques, such as splice or parabolic correction, may improve confidence in the data when combining information from separate cameras. In this case, combining the data from the two cameras still provided meaningful information without removing the jump. The spectral signatures with the jump arevstill representative of the spectral properties of the plants, providing the jumps are consistent in the calibration and validation datasets. A further consideration in combining the two datasets is the difference in the spatial resolution of the two cameras. The issue of different spatial resolutions was reduced by extracting the mean spectrum of the entire plant rather than using pixel-level information. Combining the information from two cameras gives meaningful information at the plant level but the different spatial resolutions of the cameras may prove an issue when performing pixel-level analysis.

As well as providing information related to the biochemical properties of plants, the developed prediction maps provide a visual representation of model performance. This is apparent when comparing the distribution maps of nitrogen to those of water content; the nitrogen maps appear “noisier” in comparison to the water maps. The spatial distribution and patterns within the plant do not appear feasible and may not be caused by real spatial variation in nitrogen. This is likely an effect of model overfitting (Gowen et al., 2014). This noise could be reduced by selecting a different PLSR model with which to create the maps, likely one with fewer components.

One of the main advantages of developing distribution maps is their potential to be used in time-series analyses in order to track the dynamics of biochemical parameters throughout the growing season. Images can be non-destructively acquired at multiple times throughout the season and used to predict changes or remobilisation of biochemical components over time. While hyperspectral images were acquired weekly, reference measurements of nitrogen and water were only collected at the conclusion of the experiment. Therefore, models were developed using plants of only one age (61 DAS). The growth stage of the plant has a strong influence on the developed models (Li et al., 2010; Haiying and Hongchun, 2016; Wen et al., 2019). As such, time-series analysis would first require the development of robust models using a variety of plant ages and growth stages.

Using hyperspectral phenotyping methods to estimate the content and spatial distribution of nitrogen and water in wheat shows strong promise but models could be improved by incorporating additional data from a larger range of growing conditions, e.g., seasons, soils, and genotypes. Using the techniques and methods developed here, it may be possible to measure other plant biochemical and structural properties, such as other nutrients, salts, lignin, cellulose, and water-soluble carbohydrates. Such methods may lead to advances in high-throughput phenotyping and subsequent improvements in the way that breeding trials are conducted and their biochemical properties analysed.

## Conclusion

The development of distribution maps through hyperspectral imaging was demonstrated as a nondestructive, *in vivo* tool for estimating the concentration, and spatial distribution of water content and nitrogen levels in wheat. Hyperspectral images were collected of wheat plants and multivariate regression was performed in order to relate the spectral information to the measured water and nitrogen levels. Both plant water content and nitrogen level could be predicted with “acceptable” accuracy using PLSR models developed with the mean reflectance from the full-spectra wavelengths. Wavelength selection using a regression coefficient approach slightly improved model accuracy while significantly reducing model complexity.

The regression coefficients from the best-performing VNIR models were applied to the calibrated images to develop distribution maps. The water distribution maps provided a plausible visual representation of the water distribution within the plant, however, the nitrogen maps appeared to suffer from noise likely due to model overfitting. The findings and methods from this study demonstrate the high potential of hyperspectral imagery, multivariate regression, and distribution maps have for estimating the level and distribution of plant chemical properties.

## Data Availability Statement

The raw data supporting the conclusions of this manuscript will be made available by the authors, without undue reservation, to any qualified researcher.

## Author Contributions

All authors listed have made substantial, direct, and intellectual contribution to the work and approved it for publication.

## Funding

The Plant Accelerator^®^, Australian Plant Phenomics Facility, is funded under the National Collaborative Research Infrastructure Strategy (NCRIS). BrBr acknowledges the University of Adelaide for research support through the provision of an Australian Government Research Training Program Scholarship and The Plant Accelerator^®^ for a PhD stipend top-up. Financial support from the Grains Research and Development Corporation (GRDC) and The AW Howard Memorial Trust (Department of Primary Industries and Regions, South Australia) is also acknowledged.

## Conflict of Interest

The authors declare that the research was conducted in the absence of any commercial or financial relationships that could be construed as a potential conflict of interest.
