# Supplementary material for: The Development of Hyperspectral Distribution Maps to Predict the Content and Distribution of Nitrogen and Water in Wheat (Triticum aestivum)
Source: Front Plant Sci. 2019 Oct 30;10:1380. doi: 10.3389/fpls.2019.01380 (PMC6831646; doi:10.3389/fpls.2019.01380)
Supplement: Supplementary file 1 [file Table_1.docx]

Supplementary Material

**Supplementary Table 1.** Performance results for the prediction of water content in wheat using VNIR (400-1000nm) spectra. R^2^=coefficient of determination, RMSE=root mean square error RPD=ratio of performance to deviation. PLSR= partial least squares regression, PCR= principal components regression, MLR= multiple linear regression, RF= random forest, SVM= support vector machine, ASGD1= absorbance transformation then Savitzky-Golay first derivative, ASGD2= absorbance transformation then Savitzky-Golay second derivative, CRR= continuum removal reflectance, NBR= normalization by range, EMSC= extended multiplicative scatter-correction, MSC= multiplicative scatter-correction, SGD1= Savitzky-Golay first derivative, SGD2= Savitzky-Golay second derivative, SNV= standard normal variate, SMO= smoothed. Validation R^2^≥ 0.5 are in bold.

|  |  |  |  |  |  |  |  |
| --- | --- | --- | --- | --- | --- | --- | --- |
| **Calibration** | | | | | | |  |
|  |  | PLSR | PCR | MLR | RF | SVM |  |
| ASGD1 | R^2^ | 0.63 | 0.42 | 0.50 | 0.47 | 0.84 |  |
|  | RMSE | 2.92 | 3.61 | 3.42 | 3.46 | 1.96 |  |
|  | RPD | 1.63 | 1.32 | 1.39 | 1.38 | 2.43 |  |
| ASGD2 | R^2^ | 0.57 | 0.37 | 0.35 | 0.37 | 0.90 |  |
|  | RMSE | 3.12 | 3.77 | 3.98 | 3.79 | 0.71 |  |
|  | RPD | 1.52 | 1.26 | 1.20 | 1.26 | 2.79 |  |
| NBR | R^2^ | 0.60 | 0.49 | 0.46 | 0.48 | 0.68 |  |
|  | RMSE | 3.02 | 3.41 | 3.55 | 3.45 | 2.71 |  |
|  | RPD | 1.58 | 1.39 | 1.34 | 1.38 | 1.76 |  |
| EMSC | R^2^ | 0.62 | 0.32 | 0.33 | 0.39 | 0.70 |  |
|  | RMSE | 3.03 | 3.96 | 4.29 | 3.71 | 2.72 |  |
|  | RPD | 1.57 | 1.20 | 1.11 | 1.28 | 1.75 |  |
| MSC | R^2^ | 0.62 | 0.49 | 0.45 | 0.52 | 0.66 |  |
|  | RMSE | 2.97 | 3.43 | 3.61 | 3.30 | 2.79 |  |
|  | RPD | 1.60 | 1.39 | 1.32 | 1.44 | 1.71 |  |
| SGD1 | R^2^ | 0.66 | 0.46 | 0.50 | 0.51 | 0.87 |  |
|  | RMSE | 2.86 | 3.58 | 3.38 | 3.37 | 1.89 |  |
|  | RPD | 1.67 | 1.33 | 1.41 | 1.41 | 2.52 |  |
| SGD2 | R^2^ | 0.68 | 0.18 | 0.43 | 0.46 | 0.95 |  |
|  | RMSE | 2.80 | 4.32 | 3.66 | 3.63 | 1.31 |  |
|  | RPD | 1.70 | 1.10 | 1.30 | 1.31 | 3.62 |  |
| SNV | R^2^ | 0.61 | 0.49 | 0.45 | 0.52 | 0.66 |  |
|  | RMSE | 2.99 | 3.42 | 3.64 | 3.29 | 2.78 |  |
|  | RPD | 1.60 | 1.39 | 1.31 | 1.45 | 1.71 |  |
| SMO | R^2^ | 0.64 | 0.60 | 0.61 | 0.44 | 0.67 |  |
|  | RMSE | 2.90 | 3.04 | 2.96 | 3.57 | 2.76 |  |
|  | RPD | 1.64 | 1.57 | 1.61 | 1.33 | 1.73 |  |
| Raw | R^2^ | 0.67 | 0.60 | 0.57 | 0.44 | 0.69 |  |
|  | RMSE | 2.79 | 3.04 | 3.13 | 3.55 | 2.70 |  |
|  | RPD | 1.71 | 1.56 | 1.52 | 1.34 | 1.77 |  |
| **Validation** | | | | | | |  |
|  |  | PLSR | PCR | MLR | RF | SVM |  |
| ASGD1 | R^2^ | 0.51 | 0.41 | 0.54 | 0.49 | 0.52 |  |
|  | RMSE | 3.01 | 3.30 | 2.94 | 3.06 | 2.96 |  |
|  | RPD | 1.42 | 1.29 | 1.45 | 1.39 | 1.44 |  |
| ASGD2 | R^2^ | 0.33 | 0.34 | 0.30 | 0.34 | 0.34 |  |
|  | RMSE | 3.49 | 3.45 | 4.47 | 3.46 | 3.49 |  |
|  | RPD | 1.22 | 1.24 | 0.95 | 1.24 | 1.22 |  |
| NBR | R^2^ | 0.42 | 0.42 | 0.49 | 0.42 | 0.47 |  |
|  | RMSE | 3.37 | 3.35 | 3.05 | 3.29 | 3.13 |  |
|  | RPD | 1.27 | 1.28 | 1.40 | 1.30 | 1.36 |  |
| EMSC | R^2^ | 0.34 | 0.34 | 0.40 | 0.38 | 0.35 |  |
|  | RMSE | 3.53 | 3.56 | 3.74 | 3.35 | 3.45 |  |
|  | RPD | 1.21 | 1.20 | 1.14 | 1.28 | 1.24 |  |
| MSC | R^2^ | 0.43 | 0.43 | 0.47 | 0.41 | 0.41 |  |
|  | RMSE | 3.36 | 3.35 | 3.18 | 3.29 | 3.29 |  |
|  | RPD | 1.27 | 1.27 | 1.34 | 1.30 | 1.30 |  |
| SGD1 | R^2^ | 0.46 | 0.43 | 0.52 | 0.42 | 0.49 |  |
|  | RMSE | 3.17 | 3.35 | 3.07 | 3.24 | 3.04 |  |
|  | RPD | 1.35 | 1.27 | 1.39 | 1.32 | 1.40 |  |
| SGD2 | R^2^ | 0.48 | 0.20 | 0.24 | 0.50 | 0.47 |  |
|  | RMSE | 3.10 | 3.81 | 4.56 | 3.15 | 3.15 |  |
|  | RPD | 1.38 | 1.12 | 0.94 | 1.35 | 1.35 |  |
| SNV | R^2^ | 0.33 | 0.43 | 0.47 | 0.45 | 0.41 |  |
|  | RMSE | 3.61 | 3.35 | 3.16 | 3.17 | 3.29 |  |
|  | RPD | 1.18 | 1.27 | 1.35 | 1.35 | 1.30 |  |
| SMO | R^2^ | **0.56** | **0.54** | **0.62** | 0.47 | **0.55** |  |
|  | RMSE | **2.90** | **2.88** | **2.70** | 3.11 | **2.89** |  |
|  | RPD | **1.47** | **1.48** | **1.58** | 1.37 | **1.48** |  |
| Raw | R^2^ | **0.56** | **0.54** | **0.57** | 0.49 | **0.56** |  |
|  | RMSE | **2.85** | **2.88** | **2.82** | 3.05 | **2.86** |  |
|  | RPD | **1.50** | **1.48** | **1.51** | 1.40 | **1.49** |  |

**Supplementary Table 2.** Performance results for the prediction of nitrogen content in wheat using VNIR (400-1000nm) spectra. R^2^=coefficient of determination, RMSE=root mean square error RPD=ratio of performance to deviation. PLSR= partial least squares regression, PCR= principal components regression, MLR= multiple linear regression, RF= random forest, SVM= support vector machine, ASGD1= absorbance transformation then Savitzky-Golay first derivative, ASGD2= absorbance transformation then Savitzky-Golay second derivative, CRR= continuum removal reflectance, NBR= normalization by range, EMSC= extended multiplicative scatter-correction, MSC= multiplicative scatter-correction, SGD1= Savitzky-Golay first derivative, SGD2= Savitzky-Golay second derivative, SNV= standard normal variate, SMO= smoothed. Validation R^2^≥ 0.5 are in bold.

|  |  |  |  |  |  |  |  |
| --- | --- | --- | --- | --- | --- | --- | --- |
| **Calibration** | | | | | | |  |
|  |  | PLSR | PCR | MLR | RF | SVM |  |
| ASGD1 | R^2^ | 0.61 | 0.43 | 0.44 | 0.46 | 0.83 |  |
|  | RMSE | 0.39 | 0.47 | 0.47 | 0.46 | 0.27 |  |
|  | RPD | 1.58 | 1.32 | 1.32 | 1.37 | 2.28 |  |
| ASGD2 | R^2^ | 0.57 | 0.23 | 0.30 | 0.40 | 0.90 |  |
|  | RMSE | 0.42 | 0.55 | 0.54 | 0.48 | 0.23 |  |
|  | RPD | 1.50 | 1.13 | 1.15 | 1.29 | 2.75 |  |
| NBR | R^2^ | 0.53 | 0.38 | 0.41 | 0.37 | 0.61 |  |
|  | RMSE | 0.43 | 0.50 | 0.48 | 0.49 | 0.40 |  |
|  | RPD | 1.44 | 1.26 | 1.29 | 1.26 | 1.57 |  |
| EMSC | R^2^ | 0.62 | 0.31 | 0.34 | 0.39 | 0.69 |  |
|  | RMSE | 0.40 | 0.52 | 0.52 | 0.49 | 0.36 |  |
|  | RPD | 1.56 | 1.20 | 1.20 | 1.28 | 1.72 |  |
| MSC | R^2^ | 0.54 | 0.37 | 0.42 | 0.37 | 0.59 |  |
|  | RMSE | 0.43 | 0.50 | 0.48 | 0.50 | 0.41 |  |
|  | RPD | 1.46 | 1.25 | 1.30 | 1.26 | 1.54 |  |
| SGD1 | R^2^ | 0.58 | 0.40 | 0.38 | 0.36 | 0.82 |  |
|  | RMSE | 0.41 | 0.49 | 0.50 | 0.50 | 0.28 |  |
|  | RPD | 1.52 | 1.28 | 1.26 | 1.24 | 2.22 |  |
| SGD2 | R^2^ | 0.64 | 0.26 | 0.28 | 0.33 | 0.90 |  |
|  | RMSE | 0.38 | 0.54 | 0.55 | 0.51 | 0.22 |  |
|  | RPD | 1.63 | 1.16 | 1.13 | 1.22 | 2.84 |  |
| SNV | R^2^ | 0.54 | 0.37 | 0.41 | 0.37 | 0.59 |  |
|  | RMSE | 0.43 | 0.50 | 0.48 | 0.50 | 0.40 |  |
|  | RPD | 1.46 | 1.25 | 1.29 | 1.26 | 1.55 |  |
| SMO | R^2^ | 0.56 | 0.50 | 0.53 | 0.42 | 0.60 |  |
|  | RMSE | 0.42 | 0.45 | 0.43 | 0.48 | 0.40 |  |
|  | RPD | 1.49 | 1.40 | 1.46 | 1.31 | 1.58 |  |
| Raw | R^2^ | 0.59 | 0.49 | 0.48 | 0.42 | 0.62 |  |
|  | RMSE | 0.41 | 0.45 | 0.45 | 0.47 | 0.39 |  |
|  | RPD | 1.53 | 1.38 | 1.39 | 1.32 | 1.60 |  |
| **Validation** | | | | | | |  |
|  |  | PLSR | PCR | MLR | RF | SVM |  |
| ASGD1 | R^2^ | 0.40 | 0.42 | 0.39 | 0.48 | 0.49 |  |
|  | RMSE | 0.51 | 0.50 | 0.52 | 0.47 | 0.46 |  |
|  | RPD | 1.25 | 1.28 | 1.22 | 1.38 | 1.39 |  |
| ASGD2 | R^2^ | 0.35 | 0.16 | 0.06 | 0.38 | 0.45 |  |
|  | RMSE | 0.54 | 0.60 | 0.97 | 0.51 | 0.48 |  |
|  | RPD | 1.18 | 1.07 | 0.66 | 1.27 | 1.33 |  |
| NBR | R^2^ | 0.40 | 0.40 | 0.43 | 0.36 | 0.41 |  |
|  | RMSE | 0.53 | 0.53 | 0.51 | 0.53 | 0.50 |  |
|  | RPD | 1.21 | 1.21 | 1.26 | 1.21 | 1.27 |  |
| EMSC | R^2^ | 0.34 | 0.35 | 0.32 | 0.41 | 0.40 |  |
|  | RMSE | 0.54 | 0.53 | 0.56 | 0.51 | 0.52 |  |
|  | RPD | 1.20 | 1.22 | 1.14 | 1.25 | 1.24 |  |
| MSC | R^2^ | 0.47 | 0.42 | 0.47 | 0.42 | 0.44 |  |
|  | RMSE | 0.50 | 0.50 | 0.48 | 0.50 | 0.50 |  |
|  | RPD | 1.29 | 1.28 | 1.34 | 1.27 | 1.28 |  |
| SGD1 | R^2^ | 0.43 | 0.44 | 0.38 | 0.39 | 0.46 |  |
|  | RMSE | 0.50 | 0.49 | 0.52 | 0.51 | 0.48 |  |
|  | RPD | 1.27 | 1.30 | 1.24 | 1.25 | 1.35 |  |
| SGD2 | R^2^ | 0.26 | 0.30 | 0.07 | 0.36 | 0.43 |  |
|  | RMSE | 0.55 | 0.54 | 0.91 | 0.51 | 0.48 |  |
|  | RPD | 1.16 | 1.19 | 0.71 | 1.25 | 1.33 |  |
| SNV | R^2^ | 0.43 | 0.43 | 0.45 | 0.41 | 0.44 |  |
|  | RMSE | 0.50 | 0.50 | 0.49 | 0.51 | 0.50 |  |
|  | RPD | 1.28 | 1.28 | 1.30 | 1.27 | 1.29 |  |
| SMO | R^2^ | **0.59** | **0.54** | **0.57** | 0.33 | 0.43 |  |
|  | RMSE | **0.41** | **0.44** | **0.42** | 0.52 | 0.48 |  |
|  | RPD | **1.56** | **1.47** | **1.53** | 1.22 | 1.33 |  |
| Raw | R^2^ | **0.57** | **0.52** | **0.58** | 0.33 | 0.43 |  |
|  | RMSE | **0.42** | **0.44** | **0.42** | 0.52 | 0.48 |  |
|  | RPD | **1.53** | **1.44** | **1.54** | 1.23 | 1.33 |  |
